# Identification of Phospholipids Relevant to Cancer Tissue Using Differential Ion Mobility Spectrometry

**DOI:** 10.3390/ijms252011002

**Published:** 2024-10-13

**Authors:** Patrik Sioris, Meri Mäkelä, Anton Kontunen, Markus Karjalainen, Antti Vehkaoja, Niku Oksala, Antti Roine

**Affiliations:** 1 Faculty of Medicine and Health Technology, Tampere University, 33520 Tampere, Finland; antti.vehkaoja@tuni.fi (A.V.);; 2 TAYS Cancer Centre, Tampere University Hospital, Wellbeing Services County of Pirkanmaa, 33521 Tampere, Finland; 3 Olfactomics Ltd., 33720 Tampere, Finland; 4 Centre for Vascular Surgery and Interventional Radiology, Tampere University Hospital, 33520 Tampere, Finland

**Keywords:** phospholipid, differential mobility spectrometry, field asymmetric ion mobility spectrometry, cancer

## Abstract

Phospholipids are the main building components of cell membranes and are also used for cell signaling and as energy storages. Cancer cells alter their lipid metabolism, which ultimately leads to an increase in phospholipids in cancer tissue. Surgical energy instruments use electrical or vibrational energy to heat tissues, which causes intra- and extracellular water to expand rapidly and degrade cell structures, bursting the cells, which causes the formation of a tissue aerosol or smoke depending on the amount of energy used. This gas phase analyte can then be analyzed via gas analysis methods. Differential mobility spectrometry (DMS) is a method that can be used to differentiate malignant tissue from benign tissues in real time via the analysis of surgical smoke produced by energy instruments. Previously, the DMS identification of cancer tissue was based on a ‘black box method’ by differentiating the 2D dispersion plots of samples. This study sets out to find datapoints from the DMS dispersion plots that represent relevant target molecules. We studied the ability of DMS to differentiate three subclasses of phospholipids (phosphatidylcholine, phosphatidylinositol, and phosphatidylethanolamine) from a control sample using a bovine skeletal muscle matrix with a 5 mg addition of each phospholipid subclass to the sample matrix. We trained binary classifiers using linear discriminant analysis (LDA) and support vector machines (SVM) for sample classification. We were able to identify phosphatidylcholine, -inositol, and -ethanolamine with SVM binary classification accuracies of 91%, 73%, and 66% and with LDA binary classification accuracies of 82%, 74%, and 72%, respectively. Phosphatidylcholine was detected with a reliable classification accuracy, but ion separation setups should be adjusted in future studies to reliably detect other relevant phospholipids such as phosphatidylinositol and phosphatidylethanolamine and improve DMS as a microanalysis method and identify other phospholipids relevant to cancer tissue.

## 1. Introduction

Phospholipids (PLs) are used for the structures of the cell membrane, source materials for cell signaling molecules, and as energy storages [1]. Cancer cells alter their lipid metabolism drastically to promote rapid cell proliferation and division [2]. Even in the presence of oxygen, cancer cells use aerobic glycolysis (AG) rather than oxidative phosphorylation (OP) for energy production. AG occurring in cancer cells is known as the Warburg effect, according to its finder Otto Warburg [3]. Even though AG produces far less ATP per glucose molecule compared to OP, it is a 10–100 times faster process compared to oxidizing glucose in the mitochondria, which makes energy production over a given time period comparable [3,4]. AG establishes the preservation of carbon for anabolic processes, due to it not exiting the cell as CO_2_ as in OP. Glucose is also phosphorylated to Glucose-6-phospate, which can be directed into the pentose phosphate pathway where it is used to form nucleic acids for new neoplastic cells and NADPH, which is an essential factor in de novo lipid synthesis [5]. Cancer cells produce up to 95% of fatty acids by de novo lipid synthesis [6,7]. Fatty acids are modified into PLs such as phosphatidylcholine (PC) and phosphatidylethanolamine (PE) via the Kennedy pathway for cell membrane structures [7]. Phosphatidic acid (PA) and phosphatidylinositol (PI) are used as components for cell signaling and cell migration [8]. These changes in the lipid metabolism lead to higher concentrations of PLs in cancer tissue. Higher levels of saturated PC have also been linked to the aggressiveness of breast cancer tumors [9].

The disturbed lipid metabolism of cancer enables several potential biomarkers to identify the preneoplastic changes in tissue and the recognition of lipid metabolic pathways for targeted cancer therapy. Ideally, robust lipidomic analyses of biopsies could give information of suspicious lesions that are not yet visible in histological imaging.

Mass spectrometry (MS) studies have identified PLs as prominent markers for the identification of cancer tissue from surgical smoke [10]. Ion mobility spectrometry is an analytical technique used to separate ionized molecules based on their mobility on a gas phase. Separation is dependent on the ion’s shape, size, and charge as they travel through a buffer gas under the influence of an electric field. Differential mobility spectrometry (DMS), also known as field asymmetric ion mobility spectrometry (FAIMS), is a form of ion mobility spectrometry (IMS) which can be used for in situ analyses of gas phase molecules in atmospheric pressures [11]. DMS can be coupled with a diathermy blade or a CO_2_ laser to produce analyte sample smoke, which is guided to the analysis system via a smoke evacuator. The sample analyte smoke is filtered, ionized, and subjected to an asymmetric alternating electric field where ion separation takes place. The ions hit a detector plate, losing their charge and producing electric signals which can be displayed as a 2D dispersion plot or ’smell fingerprint’.

Sample-specific dispersion plots can be identified using machine learning by training binary classifiers such as linear discriminant analysis (LDA), support vector machines (SVM), or nonlinear convolutional neural networks (CNN) [12], ultimately working as a ’black box method’ for analysis [12,13,14,15]. MS can be held as the gold standard for the analysis of biological tissues, but it has limiting factors, such as complexity, cost, and maintenance, which challenges its broad-ranging use in the medical field to analyze tissue in real time. Compared to MS, DMS is a robust method for in situ tissue analysis. DMS operates in atmospheric pressure and produces a fraction of the operating and maintenance expenses of MS [11].

The use of FAIMS has been implemented for the identification of cancer biomarkers such as proteins [16], and has been used in tandem with MS to produce more separational resolutions of potential cancer tissue biomarkers [17]. Our team has studied the microanalysis of PLs in only a single DMS study, where biologically relevant concentration increases in the PL mixture lecithin were detected in a water solution headspace with the ENVI-AMC (Environics, Finland) DMS device [18]. Identifying specific PLs distributed in a biological matrix from surgical smoke produces a continuation for DMS’ microanalysis potential and unravels the effects of ’chemical noise’ on target molecule detection. The DMS analysis system does not need sample preparation prior to the formation of surgical smoke, and a trained expert is not required to interpret the analysis results due its automated nature, thus providing rapid and uninterrupted workflow.

This study sets out to improve the spectral analysis of specific molecules relevant to the identification of cancer tissue from surgical smoke and enable a potential transition from a ‘black box method’ to a more refined microanalysis. The main objective of this study is to identify whether PL specific spikes can be identified from the dispersion plots when using a biological matrix and to study whether it is possible to identify the elevation of a single phospholipid concentration from a biological sample matrix using DMS.

## 2. Results

Sample sizes of each class were approximately similar, and corresponded to the sample sizes of our teams’ previous studies, where adequate statistical power was achieved with six classes and a relatively small sample size of 31–60 per class [19].

### 2.1. Determination of Phospholipid Detection Threshold

The detection threshold was determined by differentiating sample classes with incremental PL amounts from a zero sample with no additional PL. Results are represented in Table 1.

Table 1 shows an increase in binary classification accuracy (CA), which can be seen as the PL amount is increased. An amount of 5 mg of PC can be determined to be the cutoff for a reliably accurate detection threshold. Sample sizes were similar.

### 2.2. Binary Classification of Phospholipid Classes

Binary CAs for all PL classes with a concentration of 5 mg/g and sample sizes (n) are presented in Table 2.

As can be seen from Table 2, SVM produced high CAs for the distinctive PC samples, but LDA had less CA deviation between all PL classes. Sample sizes were identical between classes. It is shown that PC samples are more distinguishable from control samples compared to PI and PE samples.

**Figure 1:**
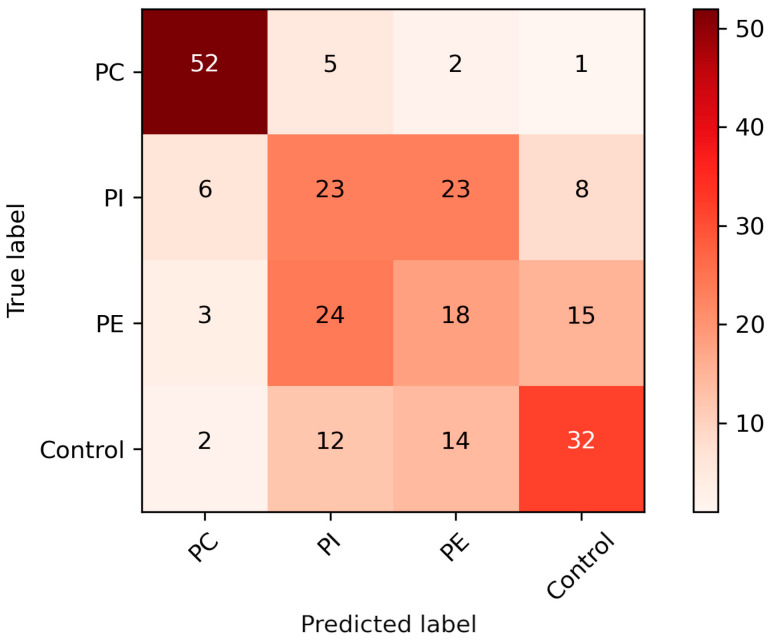
Four-class classification of all of the phospholipid classes, including the control class, using SVM. PC = phosphatidylcholine, PI = phosphatidylinositol, PE = phosphatidylethanolamine.

As can be seen from Figure 1. PC produced the greatest classification accuracy (CA), following PI and PEA, which were often misclassified as each other. PC is clearly differentiated from the control class, but PEA samples were also often misclassified as control samples.

**Figure 2:**
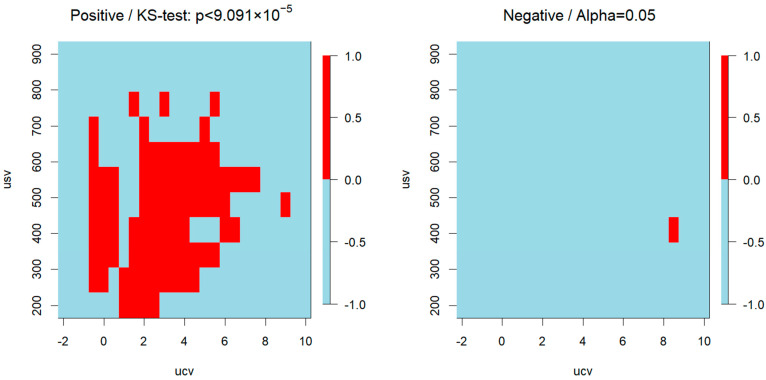
KS test and statistically significant regions of phosphatidylcholine samples.

The KS test finds statistically significant regions with regard to the class separation of the dispersion plots. Figure 2. shows that in PC samples, these regions can be seen primarily on the positive ion side. Similar results of the PI and PE sampled are discussed in the Supplementary Material S1.

## 3. Discussion

DMS has been used as an effective macro analytical method to identify malignant and benign tissues from surgical smoke. In this study, we demonstrated DMS’ ability to reliably detect small amounts of PC from a bovine skeletal muscle matrix with a CA of 83–91%, which produced information to improve the spectral analysis of cancer tissue from surgical smoke. PE and PI produced comparably inferior CAs of approximately 66–74%, so limiting factors need to be considered to improve the identification of multiple different subclasses of PLs relevant in cancer tissue.

The surgical smoke analysis of PLs from a biological matrix establishes a step for shifting from a ‘black box’ design to a more specific analysis method. This was the first microanalysis study of surgical smoke carried out with Resect™ and the Ionvision DMS-analyzer (Olfactomics Ltd., Tampere, Finland), and provided continuation for our previous study where lecithin was detected in the headspace of a water solution with concentrations ranging from 0 mg/mL to 6.2 mg/mL, resulting in a root mean square error of 0.40–0.41 mg/mL for the classification model [18]. The analysis of PLs from surgical smoke opens new insights to evaluate DMS’ strengths and weaknesses in the identification of specific target molecules and underlines areas of improvement, such as minimizing the confounding effects of the sample matrix.

Few novel studies have focused on the determination of PL amounts from cancer tissue samples. Cifkova et al. made a comprehensive characterization of human breast cancer and the surrounding normal tissue lipidome using hydrophilic interaction liquid chromatography (HILIC)–electrospray ionization mass spectrometry (ESI-MS) [20]. The 5 mg PL additions in this study only approximately compared to the difference in PL amounts between cancer and benign tissue, but the lipid profile of the margin area is distinct [21]. Azordegan et al. compared the lipid profile of the normal, margin, and tumor region of breast cancer samples and showed that the total amount of lipids in the margin area is over five-fold compared to the tumor and two-fold compared to normal tissue [21]. The DMS detection threshold for PL subgroups is potentially adequate due to total lipid amounts being amplified in the margin region, but a thorough study of the margin area’s PL profile of breast cancer tissue consisting of the determined levels of PL subgroups would provide essential information to ensure this claim.

PC is the most abundant PL in mammalian tissues [22], and the detection of biologically relevant levels of PC from surgical smoke unravels potential to acquire information of preneoplastic tissue regions. Discernible alterations in PC-derived lipid mediators manifest during tumorigenesis [23], and it has been demonstrated that when comparing 3D precancerous and invasive breast cancer spheroid models, PC and PE fractions of total lipids decrease as malignancy progresses [24], which supports the use of these PLs as markers, especially in the margin area, where tumorigenesis is active and in process. MALDI-MSI mapping of colorectal cancer showed that the PC composition differs in tumor-adjacent tissue and healthy tissue [25]. DMS’ sensitivity to detect PC could aid the identification of precancerous PC-related biochemical changes that are yet imperceptible using standard medical imaging or histological examinations. Additionally, Hilvo et al. noted that elevated PC levels in breast cancer specimens correlate to higher tumor grades and ER status [9], thus potentially providing information on pathological examination to categorize samples and evaluate the prognosis of the patient. 

The addition of a biological matrix’s ‘chemical noise’ is essential to simulate an in vivo setup. The inferior CAs of PI and PE might be due to the matrix effects causing ion suppression. PC is mostly in a positively charged or neutral state, and produces an abundance of positive ions independent of the pH value [26]. PE is primarily in a neutral or negative state, and is negative in a physiological or greater pH, and PI is mainly negatively charged, independent of the pH value [26,27]. In future PL analyses, the analyses’ setups could be adjusted to enhance the detection of a target molecule, and analyses of the negative ion side could improve the detection of PEs and PIs, while permanently neutral molecules might have adversely affected the results of this study. 

Bovine skeletal muscle is also rich in PC compared to PI and PE [28], which may adversely affect the ionization of these molecules or amplify the ionization of PCs from the samples. As can be seen from the 4-class classification (Figure 1), PE and PI were often classified, as each other which could implicate overlap in the DMS spectrum. Considering mass and surface area, PC and PE are quite similar, but in comparison, PI contains the inositol ring structure, which makes its mass and surface area greater, thus also affecting the flight trajectories of ions derived from the PI samples. Baker et al. studied the separation of PLs with DMS as a separation method prior to liquid chromatography–MS analysis and determined the fixed compensation voltage values (COV) for each PL subgroup [17]. Resect™ adjusts separation and compensation voltages independently from each other, which is called “hopping-mode DMS”, where predetermined USV and UCV pairs or areas of the dispersion plot can be scanned without needing a new analysis to start. Adjusting the DMS-hopping mode might improve the CAs of other PL subclasses to an extent.

The limitations of this study should be considered. This was the first micro analysis study carried out with an Ionvision (Olfactomic Ltd., Tampere, Finland) DMS device. PI and PE where not detectable with statistically significant reliability even with higher (10 mg) increments, which is presumably due to the matrix effects or ion separation setups. Adjustments of both should be studied further to access the detection of all relevant PLs. The matrix material was used to produce sufficient ‘chemical noise’, but the combined PL levels of the matrix and sample increments should be evened out to solve the proportional effects of the absolute PL amounts, ionization, and matrix effects on the CAs. It is worth noting that the matrix used in this study significantly differs from that of breast tissue, limiting comparisons to an in vivo tumor environment. Considering the statistical analysis, the use of a larger dataset and CNN could have produced greater classification accuracies, up to a certain limit [29].

Our future goal is to improve DMS microanalysis for the detection of multiple relevant target molecules for the detection of cancer from surgical smoke with a reliably CA and localize PL-specific ion peaks from the DMS spectrum. Statistically significant regions of dispersion plots can be used in future studies to set hopping-mode DMS Usv and Uc values to target these regions and improve identification of ions that produce the greatest separational significance for cancer samples. This produces a foundation for the more accurate detection of cancer in situ in future studies.

## 4. Materials and Methods

### 4.1. Chemicals and Reagents

We purchased high-quality PI, PE, and PC from Avanti Polar Lipids, Inc. Alabaster, AL, USA. Commercial ground bovine skeletal muscle was purchased and used as a matrix for the PLs.

### 4.2. Sample Preparation

Sample preparation was carried out on four separate days. Bovine skeletal muscle was taken out of the freezer and left to thaw. Once thawed, 1 g portions of bovine skeletal muscles were weighed on a scale with a resolution of 0.1 mg and then were moved into Petri dishes. The lipids were extracted and embedded in the matrix using fine surgical thumb forceps. The PLs were thoroughly distributed into the matrix manually using nitril gloves.

To determine the detection threshold, we prepared a control sample and four phospholipid samples by adding the most common PL, PC, found in mammalian cells in 2.5 mg increments up to 10 mg. Once the detection threshold was defined, we made two PL subclass sample sets, including the zero sample and PL samples by adding 5 mg of PI, PC, and PE into 1 g of bovine skeletal muscle. The detection thresholds for PE and PI were also assessed by preparing control, 5 mg/g, and 10 mg/g PL samples of the target PL.

### 4.3. Sampling

A monopolar surgical blade was used to produce surgical smoke from the samples. A randomized incision order was produced using a randomizer algorithm to prevent the bias of the previous measurement affecting the latter one. IN total, 30 incisions were made into each 1 g sample, combining to a total of 347 incisions for the PC detection threshold sets, 240 incisions for the four-class PL classification sets, and 180 incisions for the additional PE and PI detection threshold sets. Each incision lasted approximately 1 s. Measurements were excluded if they produced faulty dispersion plots, technical errors in the measurement system, or if the incisions produced extensive charring. The sampling deviation was visually monitored during measurements based on the live feedback of the sampling device. Significantly differing measurements were then excluded and repeated. The deviation was then additionally inspected during data analysis based on the signal intensity (pico ampers, pA) at the base of the DMS spectra. As a reference, the mean and standard deviation of the signal intensity were 180 pA and 26 pA in the PC detection threshold series and 222 pA and 31 pA in the multiple PL class detection series. An “empty” measurement of room air produces a signal intensity of 50 pA.

**Figure 3:**
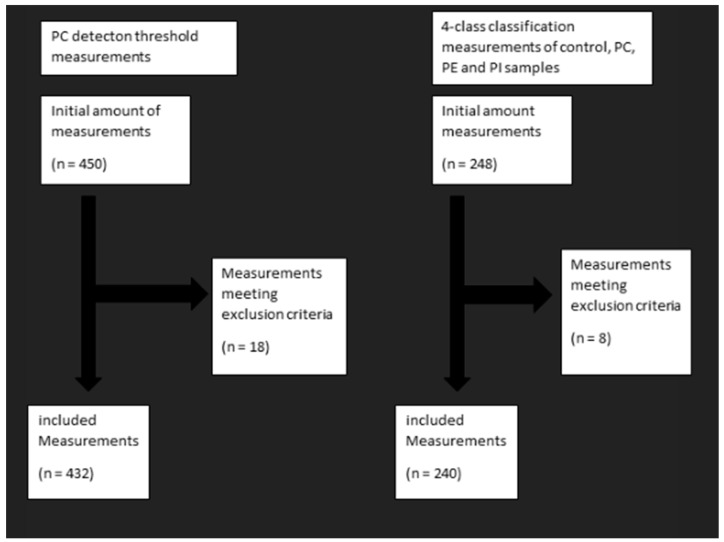
Measurement amounts (n) and excluded measurements.

A measurement met the exclusion criteria if the produced dispersion plot was visibly faulty or had technical errors such as prolonged incisions causing extensive charring and thus corrupting the quality of the measurement. Excluded measurements are presented in Figure 3.

**Figure 4:**
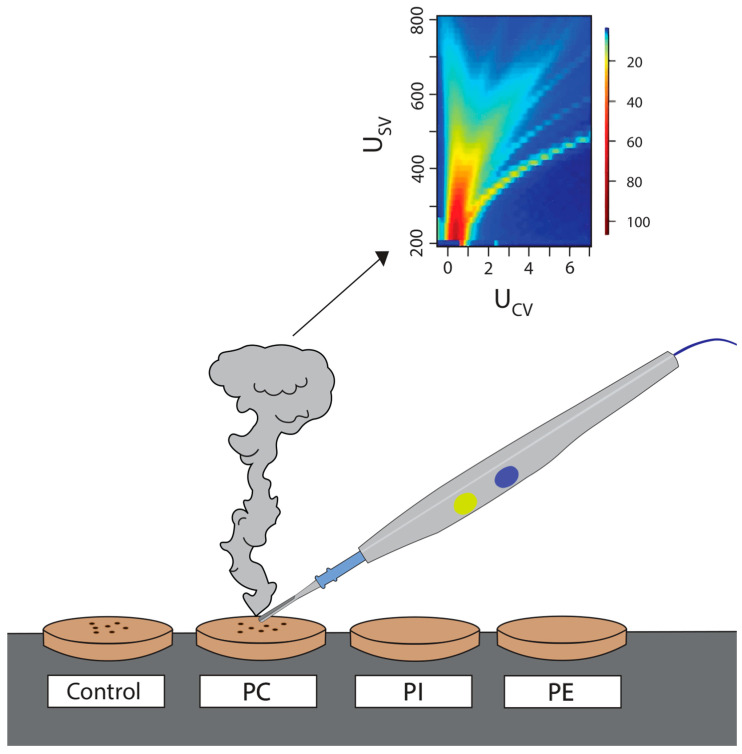
Measurements of PL samples with a diathermy blade. PC = phosphatidylcholine; PI = phosphatidylinositol; PE = phosphatidylethanolamine.

Figure 4 shows a visual representation of the tissue analysis system. The surgical smoke from each sample produces a dispersion plot, with its distinctive features enabling the identification of the sample via pattern recognition and machine learning.

### 4.4. Measurement System

In this study, we used a prototype Resect™ (Olfactomics Ltd., Finland) system, which consists of the Ionvision DMS-analysator (Olfactomics Ltd., Finland) and a sample preprocessing unit. A monopolar surgical blade Itkacut 350 MB diathermy device (Innokas Medical, Kempele, Finland) was connected to the analysis system via a *SafeAir^®^ Smoke Evacuator compact* surgical smoke evacuator (Stryker Corp, Kalamazoo, MI, USA). The system is described in detail in the study by Karjalainen et al. [30]. The diathermy power was set to 15 W. 

DMS’ ability to separate ions relies on differences in ion mobility in low and high electric fields. In DMS, the gaseous sample stream is first ionized, producing so-called reactant ions. The reactant ions then transfer their charge to the analyte molecules based on their proton or electron affinity. The higher the affinity, the easier the charge transfer reaction happens. After ionization, the sample is subjected to an asymmetric alternating electric field, controlled using separation voltage (Usv), and an orthogonal direct current electric field controlled by compensation voltage (Ucv). By altering the field strengths, different ion species can be selectively let through to the detector region, and a 2D dispersion plot can be produced. 

Resect™ can adjust the separation and compensation voltages independently from each other, which is called “hopping-mode DMS”. Predetermined USV and UCV pairs or areas of the dispersion plot can be scanned without needing a new analysis to start. In this study, the separation voltage was changed from 200 V to 900 V with 11 increments, and compensation voltage from −2 V to 10 V with 25 increments. This resulted in a dispersion plot of 275 pixels. The frequency of the separation waveform was 1 MHz, and the bias voltage was –6 V.

### 4.5. Statistical Analysis

The comparisons were performed with linear discriminant analysis (LDA) and support vector machine (SVM) models. A linear kernel was used with the SVM, and LDA used singular value decomposition (svd) as the solver. All analyses were performed in Python using the sklearn package. Leave-one-out cross-validation was used. Kolmogorov–Smirnov (KS) tests were performed between the binary groups to examine the results of the classifications. The presence of statistically differing regions in the spectra between the groups supports the validity of the classification. A Bonferroni correction was performed to the original significance level of 0.05 due to the high number of features. KS test results between the control samples and the highest concentration of PLs were plotted to highlight the areas of the DMS spectra, showing the addition.

## 5. Conclusions

Small elevations of PC can be detected with reliable accuracy from a bovine skeletal muscle matrix using DMS and localization of significant pixels of PC samples from the dispersion plot was achieved. Ion separation setups should be suited for the detection of other relevant PLs, and the sample matrix should be adjusted to mimic an in vivo tumor environment to better represent the chemical noise caused by the components of the real tumor extracellular matrix, such as laminin, collagen IV, nidogen, endothelial growth factor, and cancer-associated fibroblasts [31].

## Figures and Tables

**Table 1:** Binary classification accuracy as a function of PC content (mg/g) using linear SVM for classification and class sample sizes.

PC Amount (mg/g)	Binary Classification Accuracy (%)	Sample Size (n)
2.5	62	85
5.0	84	89
7.5	90	86
10	96	87

**Table 2:** Linear SVM and LDA binary classification accuracies (CA) of samples with 5 mg/g PL content and sample sizes (n) for each measured phospholipid class.

Phospholipid Class	SVM CA	LDA CA	Sample Size (n)
PC	91%	83%	60
PI	73%	66%	60
PE	66%	72%	60

PC = phosphatidylcholine, PI = phosphatidylinositol, PE = phosphatidylethanolamine.

## Data Availability

Data will be made available by specific request from corresponging author.

## Supplementary Materials

The following supporting information can be downloaded at: https://www.mdpi.com/article/10.3390/ijms252011002/s1.
