# Supplementary material for: Identification of Phospholipids Relevant to Cancer Tissue Using Differential Ion Mobility Spectrometry
Source: Int J Mol Sci. 2024 Oct 13;25(20):11002. doi: 10.3390/ijms252011002 (PMC11508011; doi:10.3390/ijms252011002)
Supplement: Supplementary file 1 [file ijms-25-11002-s001.zip › ijms-3227646-supplementary.pdf]

Supplementary material S1

Supplementary Table S1 Linear SVM binary classification accuracies as a function of PL content (mg/g) and sample sizes (n)

PL amount (mg/g)	Binary classification accuracy (%)	Sample size (n)
PI		
7.5	63	30
10	60	30
PE		
7.5	62	30
10	72	30

Based on the classification accuracies and the KS-test results for PEA and PI, we cannot confidently present that the 7.5 mg/g or 10 mg/g increases could be detected with the current method. It is noteworthy that the CA of PEA rose to 72 % using the 10 mg/g samples. This might be due to PEA being the second most abundant PL in skeletal muscle tissue (31), which may cause ionisation amplification of these molecules with higher PEA increments.

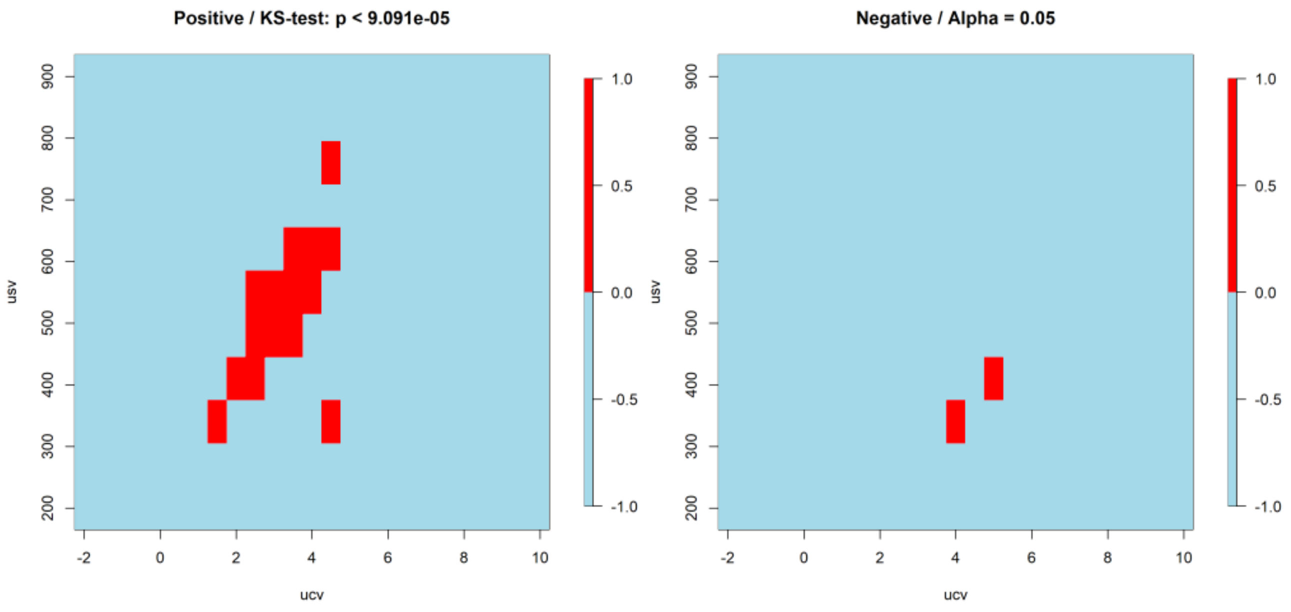

Supplementary Figure S1. KS test and statistically significant regions of PI dispersion plots

Significant regions were identified mostly from the positive ion side and also the negative. PE samples didn't produce statistically significant regions in this study.
